# Learning by doing: an observational study of the learning curve for ultrasonic fundus-first dissection in elective cholecystectomy

**DOI:** 10.1007/s00464-021-08976-z

**Published:** 2022-03-14

**Authors:** My Blohm, Gabriel Sandblom, Lars Enochsson, Yücel Cengiz, Edmunds Austrums, Elisabeth Abdon, Joakim Hennings, Mats Hedberg, Ulf Gustafsson, Angelica Diaz-Pannes, Johanna Österberg

**Affiliations:** 1 Department of Clinical Sciences, Intervention and Technology, Karolinska Institute, Stockholm, Sweden; 2 Department of Surgery, Mora Hospital, Mora, Sweden; 3 Centre for Clinical Research Dalarna - Uppsala University, Falun, Sweden; 4 Department of Clinical Science and Education, Södersjukhuset, Karolinska Institute, Stockholm, Sweden; 5 Department of Surgical and Perioperative Sciences, Surgery, Umeå University, Umeå, Sweden; 6 Department of Surgery, Central Hospital Kristianstad, Kristianstad, Sweden; 7 Department of Surgery, Östersund Hospital, Östersund, Sweden; 8 Division of Surgery, Department of Clinical Sciences, Danderyd Hospital, Karolinska Institute, Stockholm, Sweden; 9 Department of Surgery, Södertälje Hospital, Södertälje, Sweden

**Keywords:** General surgery, Laparoscopic cholecystectomy, Gallstones, Elective surgical procedures, Learning curve, Video recordings

## Abstract

**Background:**

Surgical safety and patient-related outcomes are important considerations when introducing new surgical techniques. Studies about the learning curves for different surgical procedures are sparse. The aim of this observational study was to evaluate the learning curve for ultrasonic fundus-first (FF) dissection in elective laparoscopic cholecystectomy (LC).

**Methods:**

The study was conducted at eight hospitals in Sweden between 2017 and 2019. The primary endpoint was dissection time, with secondary endpoints being intra- and postoperative complication rates and the surgeon’s self-assessed performance level. Participating surgeons (*n* = 16) were residents or specialists who performed LC individually but who had no previous experience in ultrasonic FF dissection. Each surgeon performed fifteen procedures. Video recordings from five of the procedures were analysed by two external surgeons. Patient characteristics and data on complications were retrieved from the Swedish Registry of Gallstone Surgery and Endoscopic Retrograde Cholangiopancreatography (GallRiks).

**Results:**

Dissection time decreased as experience increased (*p* = 0.001). Surgeons with limited experience showed more rapid progress. The overall complication rate was 14 (5.8%), including 3 (1.3%) potentially technique-related complications. Video assessment scores showed no correlation with the number of procedures performed. The self-assessed performance level was rated lower when the operation was more complicated (*p* < 0.001).

**Conclusions:**

Our results show that dissection time decreased with increasing experience. Most surgeons identified both favourable and unfavourable aspects of the ultrasonic FF technique. The ultrasonic device is considered well suited for gallbladder surgery, but most participating surgeons preferred to dissect the gallbladder the traditional way, beginning in the triangle of Calot. Nevertheless, LC with ultrasonic FF dissection can be considered easy to learn with a low complication rate during the initial learning curve, for both residents and specialists.

**Supplementary Information:**

The online version contains supplementary material available at 10.1007/s00464-021-08976-z.

Laparoscopic cholecystectomy (LC) is widely considered a routine surgical procedure and is expected to be part of what a surgeon under training should manage. Despite being a standardised procedure, complications are seen following elective as well as acute operations. As the safety of the procedure depends on the surgeon’s skill, it is crucial to continuously develop the surgical technique and identify potential hazards related to the equipment used.

When introducing surgical techniques or instruments, it is important to evaluate the patient-related outcomes and the surgeon’s learning curve, to avoid unnecessary complications. A learning curve reflects the development, performance and level of experience of someone acquiring a new technique or skill [1]. Studies about the learning curves for different surgical procedures are sparse and varying methodologies are used [2]. Most learning curve studies in surgery define procedure time as the primary outcome and an indirect measure of surgical skills [3]. Other procedural measures such as blood loss, surgical radicality of tumour excisions, reoperation rate, and the surgical complication rate are easily registered. However, these are all surrogate measures of patient outcome. Patient-related factors such as mortality, morbidity, length of hospital stay and quality of life are important when analysing the surgical learning curve and quality of care but may be difficult to assess with sufficient statistical power [4, 5]. Today, we have the benefit of laparoscopic surgery enabling video evaluation of the surgeon's technique in a simple way. The complicity of surgical research and implementation of new surgical techniques have been described in the innovation, development, exploration, assessment, and long-term study (the IDEAL model) [6, 7]. General quality measures in learning curve studies are still to be developed, analysed and standardised.

There is debate about the number of procedures needed to reach proficiency levels in surgery. In a 2015 review the suggested numbers, to reach a plateau phase, ranged from 25 to several hundred procedures, depending upon the complexity of the operation and previous surgical experience [3]. With extensive previous experience, the numbers decrease and may be 10 to 15 operations [8, 9].

The widely accepted method of choice in gallbladder surgery is laparoscopic access and monopolar electrocautery dissection, usually starting from the triangle of Calot. An alternative instrument for dissection is an ultrasonic device, often used with the fundus-first (FF) approach [10, 11]. This method is arguably time efficient, reduces bleeding and may result in faster recovery after LC [12–16], qualities that make the technique interesting both for improving outcomes and minimizing direct and indirect costs [17]. On the other hand, some claim that the FF approach may increase the risk of bile duct injury, especially in complicated, inflamed cases and during the early course of the learning curve [18, 19]. However, this has been disputed, and one of the first studies describing the technique noted that it might be especially beneficial in complicated cases [10]. FF dissection of the gallbladder with harmonic scalpel is also used in more extensive abdominal procedures, such as minimally invasive duodenopancreatectomy [20, 21]. The ultrasonic device differs from the traditional method with electrocautery and practice is needed to optimize the instrument handling. One centre in Sweden uses the ultrasonic FF technique as their standard method, showing good results and a low complication rate [12, 13]. The results have been mainly attributed to the ultrasonic instrument, and not the FF dissection [12]. This has aroused interest in the technique and in ultrasonic dissection, either FF or from the triangle of Calot. It is considered a useful alternative, especially in acute and chronic cholecystitis where the instrument’s vessel-sealing capacity might be very useful. In Sweden in 2019, 6.8% of all elective LCs were performed with FF dissection [22].

The aim of this study was to evaluate the learning curve for ultrasonic FF dissection in elective LC, focusing on the dissection time and surgical safety, in terms of the intra- and postoperative complication rates. If the technique proves to be easy to learn with a low complication rate during the initial learning curve, it might stand as an alternative to electrocautery dissection and be a way to further improve surgical safety and patient-related outcomes. The study was also intended as pilot study for a randomized controlled trial, comparing LC with monopolar electrocautery to ultrasonic dissection, in patients with acute cholecystitis.

## Materials and methods

### Study design

The study was designed as a multicentre observational study of the learning curve for ultrasonic FF dissection. The primary endpoint was dissection time. Secondary endpoints were intra- and postoperative complication rates and the surgeon’s self-assessed performance level. Procedures were included from May 1st, 2017 to December 31st, 2019. Data were collected from a procedure-related case report form (CRF), video recordings and the Swedish Registry for Gallstone Surgery and Endoscopic Retrograde Cholangiopancreatography (GallRiks). This registry includes a 30-day postoperative follow-up based on medical records, registered by a local coordinator at each participating hospital. All participating surgeons answered a final questionnaire regarding the technique. The study report was structured in accordance with the STROBE (*Strengthening the Reporting of Observational Studies in Epidemiology*) reporting checklist [23]. The study is registered on ClinicalTrials.gov: (NCT03154164).

### Participants

Twenty-one surgeons from nine Swedish hospitals participated and undertook fifteen procedures each. All surgeons were able to perform LC independently with the traditional monopolar electrocautery approach but had no previous experience of ultrasonic FF dissection. Before the first procedure, the participants attended a day-long mandatory surgical education session that included both lectures and real-time observation of live operations. The surgeons were categorised as resident or specialist, based on their position at the start of the study. The estimated number of previously conducted cholecystectomies, as well as previous experience of the ultrasonic instrument in other surgical settings, were recorded. At a minimum, the assistant had to be at least a surgical resident. The study period for each individual surgeon was not defined beforehand, for practical reasons.

The study included patients without laboratory or radiological signs of acute or chronic cholecystitis who were scheduled for an elective cholecystectomy. Patients who were unable to understand information about the study and give consent, patients aged < 15 years, and patients with a preoperative diagnosis of acute cholecystitis or choledocholithiasis, were excluded. All included patients signed a written informed consent form before the procedure. Final decisions on inclusion were made intra-operatively, if no macroscopic signs of active or chronic cholecystitis were confirmed before starting the dissection.

### Surgery

**Fig. 1:**
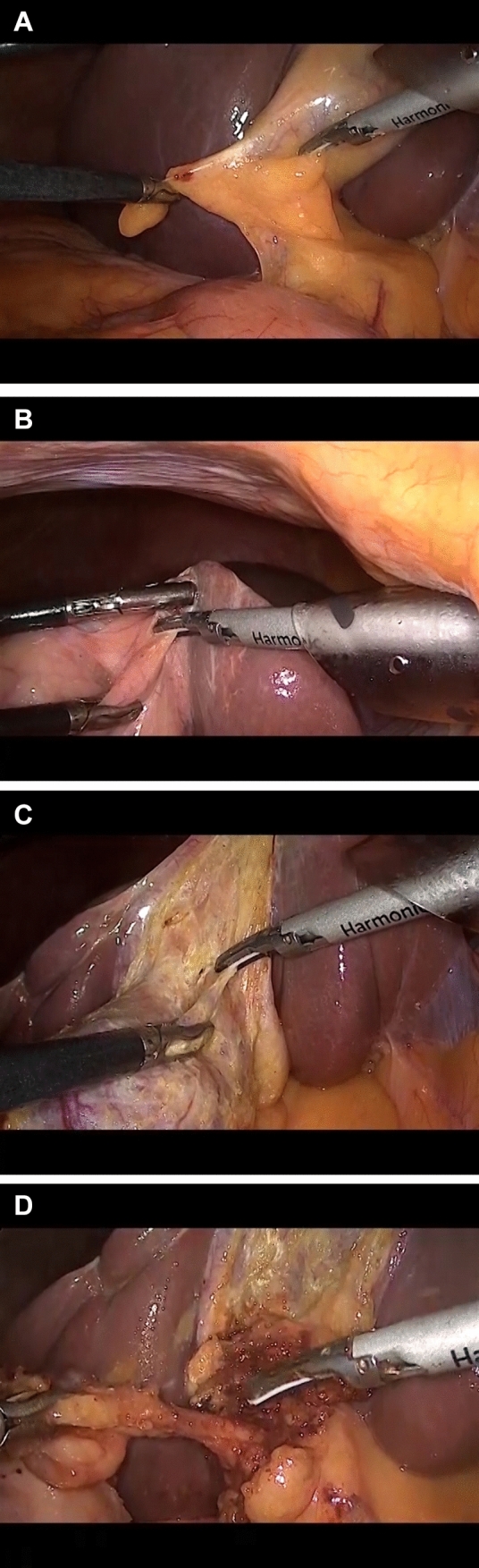
**A–D** The ultrasonic fundus-first dissection. **A** The dissection starts by marking the peritoneum where the surgeon estimates that the gallbladder continues into the cystic duct. **B** The assistant places a grasper on the peritoneum between the top of the gallbladder and the liver. The dissection then begins, and the correct space is identified. **C** The dissection continues, alternating on the medial and lateral sides, until the already-defined marking is reached. **D** The artery and cystic duct in the triangle of Calot are cleared and visualized. A cholangiography is performed, and the artery and cystic duct are divided by using at least two proximal clips

The ultrasonic instrument used in the study was Harmonic^®^ ACE + (Ethicon Endosurgery (Europe) GmbH, Norderstedt, Germany). To enable comparisons and analyses of time and video recordings, the surgeries followed a standardised protocol. Anaesthesia was conducted according to local routines for elective cholecystectomy. An open-entry technique (Hasson) below the umbilicus was used to access the abdomen followed by placement of the first trocar. In complex cases, a Veress needle could be used just below the left subcostal arch at Palmer´s point together with the optical trocar under the umbilicus. The intra-abdominal pressure was kept at 12 mmHg or, if needed, a maximum of 16 mmHg in obese patients. A standard four-port setting was used. The gallbladder was inspected and a final decision concerning study inclusion was made. The ultrasonic instrument was set at level 3/5. A grasper or a liver retractor was inserted in the most lateral port to exert traction on the gallbladder. The dissection started by marking the peritoneal margin between the gallbladder and ductus cysticus before the FF dissection was initiated. Dissection continued until a “critical view of safety” was obtained. An intraoperative cholangiography was performed, according to the standard routine in Sweden, and the cystic duct was divided between clips, with at least two clips remaining on the proximal end. The cystic artery was optionally divided between clips or by the ultrasonic device, depending on the diameter of the vessel. A retrieval bag was used for gallbladder extraction through the umbilical port. Local anaesthetics were administered at all incision sites. Postoperatively, all patients were treated according to local routines. Figure 1 and video 1 (supplementary information) illustrates a standard operation with ultrasonic FF dissection according to the study protocol.

### Variables

Each surgery was divided into six different procedural steps (Table 1) and the time for each step was registered intra-operatively. Dissection time with the ultrasonic device was the primary outcome in our analysis. A numerical scale from 1 to 100 was used to grade the perceived level of difficulty (1 = very easy and 100 = very difficult) and performance level (1 = poor and 100 = excellent) for each procedural step. Patient characteristics, presence of cholecystitis, accidental perforation of the gallbladder and intra- and 30-day postoperative complications were retrieved from GallRiks.

**Table 1:** Time intervals and procedural steps

1	Start	Time from skin incision to the marking of the peritoneal margin between the gallbladder and the cystic duct, including dissection of adherences
2	Dissection	Dissection of the gallbladder according to fundus-first, from the marking of the peritoneal margin until the first clip, before the cholangiography
3	Cholangiography	Cholangiography, followed by division of ductus cysticus and arteria cystica
4	Finish	Removal of remaining fluid and bile, extraction of the gallbladder and closure of the fascia and skin incisions
5	Other	Other procedures, e.g. intraoperative ERCP, repair of umbilical hernia, etc
6	Total	Total time

### Video recordings

Videos from procedures 1, 5, 10, 14 and 15 were saved and coded, to de-identify the operation sequence number, surgeon and hospital. The films were independently assessed by two out of three external surgeons with extensive clinical experience of the FF technique. One common grading system was organized before the start of the assessment to increase concurrence. The assessors focused on the dissection step (*n* = 2 in Table 1), with pre-defined start and stop times. Each minute of the dissection was carefully viewed and graded according to the previously published error definitions defined by Seymour (2002) (Table 2) [24, 25]. The assessors also graded the level of difficulty and could leave their comments on the surgical technique. The individual scores and comments were given to each participating surgeon as feed-back after their inclusion in the study was terminated.

**Table 2:** Error definitions by Seymour

1	Lack of progress	No progress made in excising the gallbladder for an entire minute of the dissection
2	Gallbladder injury	A gallbladder wall perforation with or without leakage of bile
3	Liver injury	A liver capsule and parenchyma penetration, or capsule stripping with or without associated bleeding
4	Incorrect plane of dissection	The dissection is conducted outside the recognized plane between the gallbladder and the liver
5	Burned non-target tissue	Any application of the instrument to non-target tissue. A slight whitening of the liver capsule due to indirect vaporisation is accepted
6	Tearing tissue	Uncontrolled tearing of tissue with the dissecting or retracting instrument
7	Instrument out of view	The dissecting instrument is placed outside the field of view such that its tip could potentially be in contact with tissue, and not caused by a sudden camera movement

### Assessment of potential bias

In order to limit variations in operating and dissection times, a standardised operation protocol was used. The mandatory training included information about the time intervals, and the study protocol was presented to all participating surgeons. The CRF and GallRiks registration were completed online immediately after the surgery to reduce recollection bias. The validity of data in GallRiks is monitored regularly by independent reviewers. This registry has proven to be complete and correct, and serious complications are always reported [26]. The videos were assessed by external surgeons with clinical experience of the FF technique and previous participation in research studies with video assessments.

### Sample size

The sample size of fifteen operations per surgeon was based on the surgeons’ previous experience of gallbladder surgery. As the anatomy in most cases is uniform, the main innovation was assumed to be the ultrasonic instrument and the FF dissection. The number of surgeons was based on our aim to have participants from different hospitals and an equal distribution of previous LC experience.

### Statistical analysis

The results from intra- and postoperatively registered data, GallRiks and video recordings were matched by an individual study ID. Statistical analysis was performed with SPSS^®^ software (IBM SPSS Statistics for Windows, Version 26.0. Armonk, NY: IBM Corp). Surgeon and patient demographics were presented in contingency tables. Logarithmic transformation of times was used for the individual line charts, to make the plots more comprehensible. The association between procedural number and dissection time was analysed with multivariable linear regression modelling, adjusting for the level of difficulty, cholecystitis, gender, age and BMI of the patient. This was presented as unstandardised B, *p* values and 95% confidence intervals. We used the surgeons’ rating of the level of difficulty since the external assessment only included one third of the procedures. Inter-observer reliability of the video assessment was tested with intra-class correlation between the two independent assessors’ value of the separate error definitions, as well as the total score. Spearman’s correlation was used to assess the possible correlation between the error definition score from the video recordings with the operation number. *p* < 0.05 was considered significant.

## Results

### Participants

**Fig. 2:**
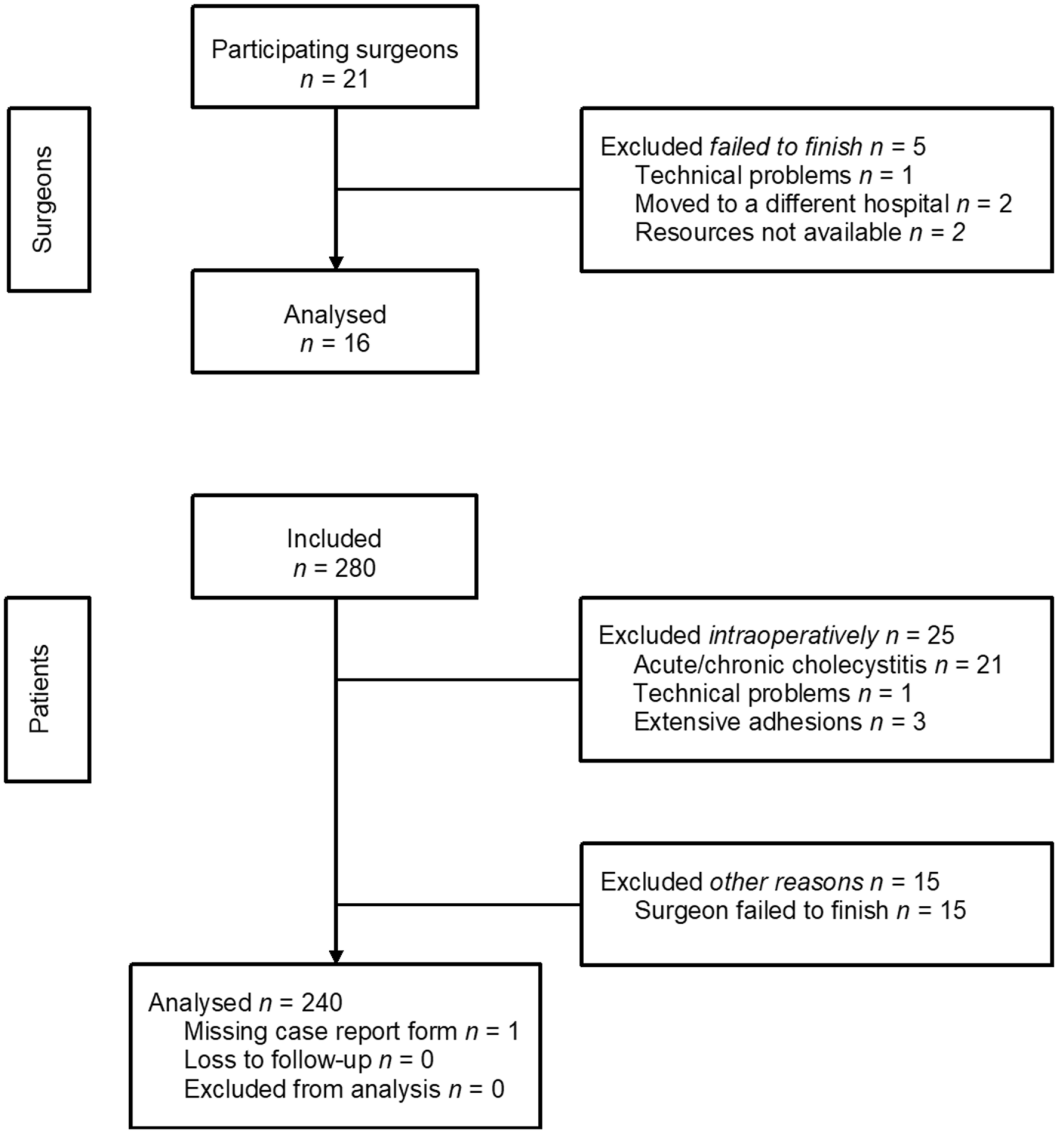
Flowchart of included and excluded surgeons and patients

Of the 21 surgeons, 16 completed 15 operations each. Five surgeons from four different hospitals dropped out early and failed to finish. In total, 240 operations were analysed. A flowchart of included and excluded patients is presented in Fig. 2. The demographics of the participating surgeons and patients are presented in Table 3.

**Table 3:** Demographics of participating surgeons and patients

Surgeons	Resident	Specialist
Years in profession		
< 5	5	0
5–20	0	7
> 20	0	4
Sex		
Female	3	4
Male	2	7
Previous cholecystectomies		
< 100	3	2
101–500	2	4
> 500	0	5
Previous ultracision use		
< 50	5	8
50–100	0	1
101–500	0	2

Patients	*n*	%
Age		
< 50	122	50.8
≥ 50	118	49.2
Sex		
Female	169	70.4
Male	71	29.6
BMI		
< 25	54	22.5
25–29	98	40.8
30–34	50	20.8
≥ 35	24	10.0
Missing	14	5.8
ASA		
1	103	42.9
2	117	48.8
≥ 3	20	8.3

### Outcome data

#### Dissection time

**Fig. 3:**
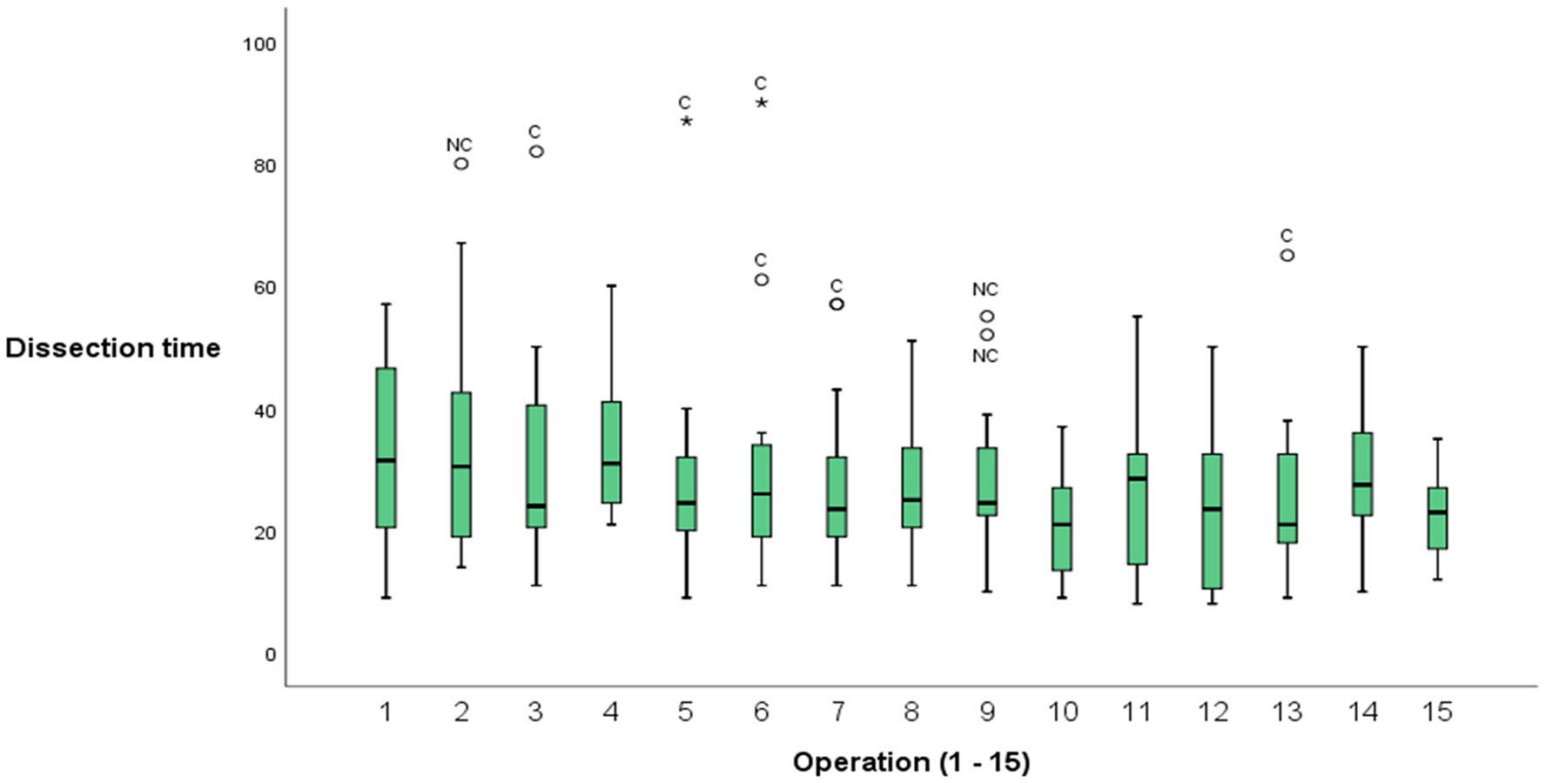
Boxplot of dissection time (min) and procedural number for all participants. Outliers (open circle) and extreme outliers (asterisk) defined as cholecystitis (C) and no cholecystitis (NC)

**Fig. 4:**
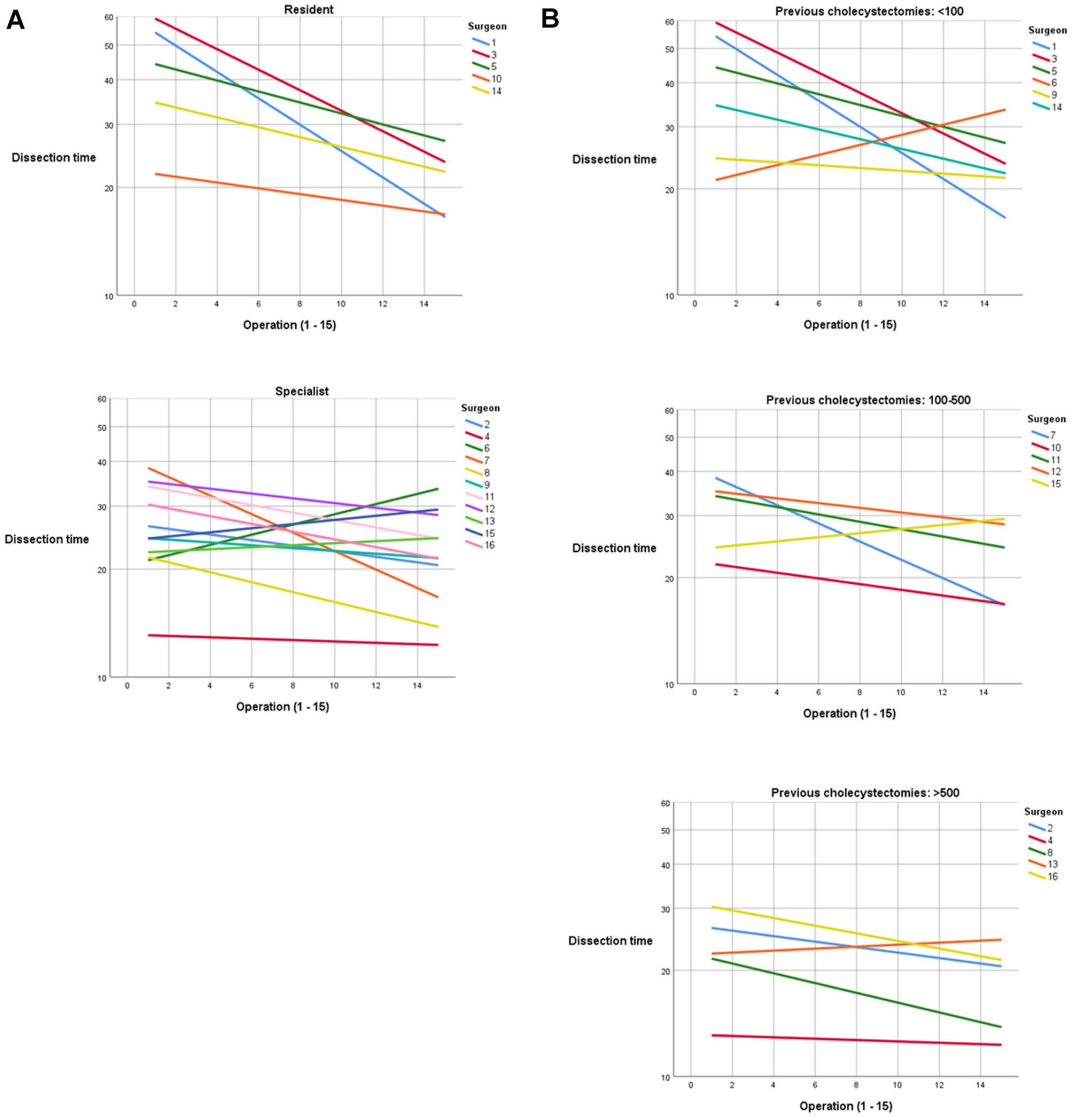
Individual learning curves of dissection time (min) for residents and specialists **A** and for surgeons with varying experience **B**

Dissection time decreased over the fifteen operations (Fig. 3). This was significant in both univariate and multivariate analysis (Table 4). The total operating time also decreased over time (*p* < 0.001). On the individual level, 14 out of 16 surgeons showed progress in shortening the dissection time and a negative correlation coefficient (data not shown). This progress was more pronounced for residents and surgeons with more limited experience. The logarithmic times are presented in Fig. 4a and b. The mean dissection time for the first five operations was 40.6 min (SD 21.4) for residents and 28.3 min (SD 11.8) for specialists compared to 25.4 min (SD 12.3) and 25.3 min (SD 11.7) for their last five procedures. The distribution of cholecystitis and other risk factors for a longer operating time, such as male gender and obesity, were equally distributed among the groups. Only three surgeons had previous experience of > 50 operations with ultrasonic dissection, mainly those with bariatric surgery experience. This had a significant impact on dissection time in the univariate analysis but not in the multivariate analysis (Supplementary Table 1).

**Table 4:** Univariable and multivariable linear regression analysis with dissection time as outcome

	Univariable		Multivariable	
	*B* (95% confidence interval)	*p*	*B* (95% confidence interval)	*p*
Number of procedure (1–15)	− 0.715 (− 1.129 to − 0.302)	0.001	− 0.481 (− 0.815 to − 0.148)	0.005
Difficulty of procedure	0.384 (0.321–0.447)	< 0.001	0.329 (0.260–0.398)	< 0.001
Cholecystitis	13.622 (9.677–17.567)	< 0.001	6.975 (3.353–10.598)	< 0.001
Gender (patient)	2.719 (− 1.263–6.702)	0.180	0.712 (− 2.475–3.900)	0.660
Age (patient)	− 0.001 (− 0.32–0.29)	0.941	− 0.005 (− 0.028 to 0.018)	0.654
BMI ≥ 30 (patient)	3.244 (− 0.782–7.271)	0.114	1.863 (− 1.214–4.940)	0.234

#### Complication rate

A crucial measure of safety during the learning curve is the intra- and postoperative complication rate. In our study the total complication rate was 14 (5.8%) with 3 (1.3%) potentially technique-related complications. Two patients showed signs of postoperative bile leakage from the cystic duct and underwent re-exploration and abdominal drainage. In both cases the cystic duct had been closed with two proximal clips. One patient underwent re-exploration due to postoperative pain, with no specific cause being identified. One intra-operative complication, a myocardial infarct, was registered (0.4%). Intraoperative cholangiography was successfully performed in 95% of the operations and stones were visualized in the common bile duct in six patients (2.5%). The stones were removed with intraoperative ERCP (3 patients), postoperative ERCP (1 patient), left without intervention (1 patient) and manipulated into the duodenum (1 patient). Consequently, one case of post ERCP pancreatitis after the postoperative ERCP and one case of postoperative pancreatitis due to stone manipulation were noted. The remaining eight complications were two urinary tract infections, one postoperative wound infection and five patients with unspecific postoperative symptoms of infection. No conversion to open surgery was noted. The technique-related complications were caused by specialists, two with experience from > 500 operations. One of these three patients had chronic cholecystitis. The complications were evenly distributed across the 15 procedures in the study and no significant association to the procedural number could be seen (*p* = 0.612).

#### Performance level and video assessment

The self-assessed performance level was generally lower in cases of a more complicated procedure (*p* < 0.001). This correlation remained when adjusting for ongoing cholecystitis (*p* < 0.001). The surgeon’s assessment of the level of difficulty corresponded to the external grading from the video assessments (intra-class correlation coefficient 0.68, *p* < 0.001). Seventy videos were analysed. Ten videos were missing due to technical problems with the recordings. This problem was mainly at the beginning of the study and, as a consequence, five (30%) of the films from the first operation were not analysed. The remaining missing films were more evenly distributed across the fifteen operations. The videos were analysed based on the error definitions. The intra-class correlation coefficient was high for gallbladder perforations (0.943, *p* < 0.001) and significant for lack of progress (0.591, *p* < 0.001), incorrect plane of dissection (0.517, *p* = 0.001) and the total score (0.655, *p* < 0.001). The remaining four categories showed no significant concurrence. No significant association could be seen between the error definitions in the video grading and the number of procedures performed by the surgeon in the study.

### Other analysis

Dissection time ranged from 8 to 90 min (mean 28 min), depending on difficulty and the presence of cholecystitis. Although the presence of acute or chronic cholecystitis was an exclusion criterion, and the gallbladder was carefully inspected before final inclusion in the study, the posterior wall of the gallbladder showed clinical signs of inflammation in 57 (23.8%) of the cases. This inflammation was defined as acute in 3 (1.3%) and chronic in 51 (21.3%) patients. The mean time between the first and last operation was 312 days (range 74 to 565 days). The time periods between the different operations were analysed in relation to the dissection time, without significant effect (*p* = 0.282). One hundred and sixty-two patients (67.5%) could be treated in outpatient care; 67 (27.9%) stayed overnight; 7 (2.9%) stayed for two nights, and the remaining four patients had a longer hospital stay (range 6 to 14 days), related to either complications or comorbidities.

An iatrogenic gallbladder perforation was noted in 73 (30.4%) of the procedures (33.3% residents, 29.1% specialists). Twenty-three (31.5%) of these had an ongoing cholecystitis. Perforations were most commonly seen on the medial side relatively close to the top of the gallbladder. Based on the video assessment, the perforation occurred early, in mean after 9 min of the start of the dissection (which includes the time for the marking of the peritoneum). Most of all perforations, 35 (47.9%), occurred when the assisting surgeon had performed < 50 cholecystectomies. No difference was seen between the remaining experience categories. Increased previous experience with the ultrasonic device did not affect the level of perforations. No significant association between gallbladder perforations and the procedural number was seen (*p* = 0.219).

## Discussion

### Key results

In this study, we evaluated the ultrasonic FF dissection technique in elective LC, with dissection time as a surrogate measure of the learning curve. Our results show that dissection time decreased significantly during the first 15 operations. This is especially evident for surgeons with more limited experience of traditional electrocautery dissection. Until now, the use of operation time as a surrogate measure of surgical skill has been the most frequently used measure in learning curve studies [2]. However, it is insufficient as the only measurement, as shown in previous studies [2, 4, 5, 28, 29].

Based on our results, ultrasonic FF dissection can be considered easy to learn and safe, with a low complication rate, during the initial learning curve for surgeons with previous experience of gallbladder surgery with monopolar electrocautery. The complication rate of 5.8% is comparable to the Swedish national complication rate for all elective LCs in 2019 (7.3% postoperative and 1.5% intraoperative complications) [22]. The two postoperative bile leakages from the cystic duct might be explained by a lack of experience of the ultrasonic device. One of the videos illustrated a tendency that the surgeon did not use enough countermovement with the grasper and thereby inserted the device into deeper structures in a dangerous way.

We decided to use the FF approach in the study since it is the dissection method typically used with the ultrasonic instrument in gallbladder surgery in Sweden. Since we focused on ultrasonic FF dissection, we are not able to distinguish whether the measured data depends on the FF dissection or the use of the ultrasonic device. One argument against the FF approach is that it may lead to overly extensive central dissection medially [18, 19]. One way to decrease this risk is to start the dissection by marking the peritoneal margin between the gallbladder and cystic duct, on both the lateral and medial sides. We recommended this in our study as an additional safety measure. Another common argument against the technique is the higher instrument cost. However, total direct and indirect costs have previously been shown to be lower with ultrasonic FF dissection compared to traditional electrocautery dissection [17].

### Other findings

We considered it important to minimize the risk of including patients with cholecystitis, seeing that cholecystitis is a known risk factor for a more complicated surgery as well as longer operating time [30, 31]. However*,* one fifth of the patients in our study had chronic cholecystitis of the posterior wall. This reflects reality since many patients have had a previously episode of unrevealed cholecystitis or frequent pain episodes. The observed perforation rate of 30% is regarded as rather high and most surgeons also noticed an increased perforation risk at the beginning of the dissection. This contradicts other studies where ultrasonic dissection is associated with a low perforation rate, compared to electrocautery dissection [12, 16, 32]. The perforation often occurred early during the most apical dissection in the fundus region. An experienced assistant can facilitate the process for the operating surgeon by indicating the correct plane with an active apical countermovement of a grasper or liver retractor. GallRiks data from recent years (2017–2019) show no significant difference in perforation rates between traditional dissection from the triangle of Calot (25.1%) and fundus-first dissection (25.1%) (*p* = 0.4328) [22].

### Clinical experience

In the final evaluation, most surgeons in our study still desired more extensive dissection in the triangle of Calot, in order to make a critical view of safety [33], before starting the FF dissection. A drawback of the technique is that it can be difficult to maintain an appropriate amount of counter tension when dissecting the cystic duct. The gallbladder may also rotate, making it difficult to insert a catheter and perform an intraoperative cholangiography, which is the clinical routine in Sweden [34]. We used the Ultracision Harmonic ACE+, which some surgeons considered too blunt, especially when dissecting the important structures in the triangle of Calot. Since the study started, newer versions with a slim and slightly curved tip have been introduced (Harmonic^®^ HD 1000i Shears, Ethicon Endosurgery (Europe) GmbH, Norderstedt, Germany).

One advantage for the FF dissection is that one obtains the optimal critical view of safety, since the gallbladder, cystic duct and cystic artery are the only remaining structures. Some surgeons described it as mentally comforting that the operation was more or less completed after the cholangiography. Most surgeons noticed an increased familiarity with and confidence in ultrasonic instrument handling during the study. The instrument has an effective vessel-sealing capacity, and the shared opinion is that it reduces bleeding in uncomplicated as well as complicated cases. It is, however, important to find the right anatomical plane between the gallbladder and liver to avoid a gallbladder perforation or bleeding from the liver. When this plane is found, the procedure is generally fast, dry and efficient. A timesaving factor is that the surgeon does not have to change instruments as frequently as with electrocautery, which is often combined with a grasper and a suction device. Other advantages include the ability to swiftly dissect and divide adherences, as well as improved ergonomics because one does not have to work with a foot pedal. The overall opinion in our study is that the ultrasonic device is well suited for gallbladder surgery, but most surgeons prefer to begin by dissecting the important structures in the triangle of Calot. The fundus-first dissection is not considered better than the traditional technique but might be useful as an alternative method in certain cases.

### Strengths and limitations

One strength of our study is that it was a multicentre study with participating surgeons from different parts of Sweden, from universities as well as regional and community hospitals. The surgeons’ previous experience of gallbladder surgery with the traditional method was equally distributed. GallRiks data were complete concerning patient characteristics, cholecystitis, perforation rate and complications. All films were video recorded which is considered a strength, even if some films were missing from the analysis due to technical problems. Although other grading systems exist, such as the Global Operative Assessment of Laparoscopic Skills (GOALS) [35], our video assessors used the error definitions defined by Seymour (2002) [24, 25] since two out of three surgeons had previous experience with these definitions. The study was an incentive for routine video recording of laparoscopic procedures at the participating clinics, thereby adding an additional surgical safety measure.

A limitation of our study is that it is an observational study with outcomes based on subjective assessments, where potential bias must be taken into consideration. Comparing the surgeons’ estimates of performance and difficulty is complicated since they are based on personality and previous experience. The video assessors’ personal technique and preference undoubtedly affected the score. The information concerning previous experience with cholecystectomies and ultrasonic dissection is based on the surgeon’s own evaluation, which might be incorrect. Another limitation is that the time between the first and 15th operation was not specified beforehand. The mean time interval was 312 days. The time spent was affected by practical issues such as reduced elective surgeries, parental leave, etc. This indicates the difficulty of organising a multicentre study on a surgical learning curve, in a clinical setting. Paradoxically, the surgeons with the two shortest intervals were the surgeons without a negative curve inclination. One explanation is that they may have been more eager to include patients in order to finish their inclusion in the study rapidly, and thus may also have included more complicated cases. The surgeon with the shortest time interval had a relatively high frequency of patients with unsuspected cholecystitis. We decided to include 15 operations per surgeon. It is possible that an increased number of procedures could have yielded a flattened curve even for residents.

### Generalizability

Our study was conducted in Sweden and based on the Swedish population and surgical setting, which affects its generalizability. However, the visualisation of the learning curve is illustrative, and the results may be applied to countries with similar routines and educational structures.

## Conclusion

Our findings imply that dissection time decreases with increased experience, especially for surgeons with less extensive previous experience of gallbladder surgery. Ultrasonic FF dissection has a low complication rate during the surgeon’s first fifteen operations and can be used as an alternative to monopolar electrocautery dissection even during the initial learning curve. Nevertheless, comparative studies, in particular randomized controlled studies (RCT), are needed to prove whether ultrasound dissection is superior to conventional dissection using electrocautery devices. Even if ultrasonic FF dissection does not become the future standard technique, it is important to have at least one alternative approach to apply in more complicated cases. We intend to evaluate this in an RCT, comparing LC with ultrasonic dissection to monopolar electrocautery, in patients with acute cholecystitis.

## Supplementary Information

Below is the link to the electronic supplementary material.

Supplementary file1 (DOCX 29 kb)

Supplementary file 2 A standard operation with ultrasonic fundus-first dissection according to the study protocol (AVI 754150 kb)
