# Supplementary material for: Learning by doing: an observational study of the learning curve for ultrasonic fundus-first dissection in elective cholecystectomy
Source: Surg Endosc. 2022 Mar 14;36(6):4602–13. doi: 10.1007/s00464-021-08976-z (PMC9085702; doi:10.1007/s00464-021-08976-z)
Supplement: Supplementary file 1 — Supplementary file1 (DOCX 29 kb) [file 464_2021_8976_MOESM1_ESM.docx]

**Supplementary Table 1. Univariable analysis**
